# Analysis of Factors Affecting Child Nutrition in Nepal Using the Nepal Demographic and Health Survey 2022

**DOI:** 10.7759/cureus.102248

**Published:** 2026-01-25

**Authors:** Seeta Baral, Minato Nakazawa

**Affiliations:** 1 Public Health, Kobe University Graduate School of Health Sciences, Kobe, JPN

**Keywords:** child nutrition, child under-nutrition, demographic and health surveys, feeding practices, six to twenty-three months children, stunting, underweight, wasting

## Abstract

Background

Child malnutrition remains a major public health problem globally, including in Nepal. Previous studies reported that low maternal body mass index, maternal education, wealth status, anemia, low birth weight, etc., are significant predictors of childhood under-nutrition. However, child feeding practices/complementary feeding, in multivariable models specific to each anthropometric outcome, have not been explored. This study, using updated information from the Nepal Demographic and Health Survey (NDHS) 2022, will address child factors such as feeding practices, especially focusing on complementary feeding of younger children, along with other birth factors, parental factors, and socioeconomic factors.

Methods

This study used nationally representative data from the NDHS 2022, where a total of 629 children aged 6-23 months were selected for analysis. Three anthropometric indicators (stunting, wasting, and underweight) were used to monitor the nutritional status of children. Independent variables were child characteristics, infant and young child feeding practices, parental factors, household wealth, and region. Binomial logistic regression analysis was carried out after adjusting for covariates. Adjusted odds ratios (ORs) with 95% confidence intervals (CIs) were reported, and p-values <0.05 were considered statistically significant.

Results

Children with increasing age (12-23 months) had significantly higher odds of stunting. Children from households with better economic status showed lower odds of stunting. Children perceived as very small birth size had significantly higher odds of stunting (OR = 6.06; 95% CI: 2.05-17.91; p = 0.001) and being underweight (OR = 6.28; 95% CI: 2.06-19.17; p = 0.001). Maternal factors showed a strong association with all three outcomes. Children whose fathers underwent secondary and higher education had significantly greater odds of wasting compared with those whose fathers had no education (OR = 3.56; 95% CI: 1.08-11.82; p = 0.038), (OR = 11.40; 95% CI: 2.14-60.58; p = 0.004), respectively. Feeding frequency and minimum dietary diversity score were not significantly associated with any of the three anthropometric outcomes.

Conclusion

The findings of this study showed that the increasing age of children was significantly associated with stunting. Maternal education, maternal age, and maternal BMI were important protective determinants of child under-nutrition. From the findings of this study, complementary feeding practices, along with maternal and household factors, may prevent child under-nutrition.

## Introduction

Good nutrition sets children on the path to grow, develop, learn, and reach their full potential. Despite significant progress over the past two decades, child malnutrition still remains a major problem worldwide. In 2024, among children under the age of five years worldwide, 23.2% (150.2 million) experienced stunting, 6.6% (42.8 million) experienced wasting, and 5.5% (35.5 million) were overweight. Nearly two out of five children with stunted growth and more than half of all children affected by wasting live in South Asia, and most children categorized as overweight live in Asia and Africa [1]. Sustainable Development Goal (SDG) 2 aims to end hunger, achieve food security and improved nutrition, and promote sustainable agriculture by 2030. But the triple burden of malnutrition - under-nutrition, hidden hunger, and overweight - adversely affects the survival, growth, and development of children and young people [2].

Although Nepal has made significant progress in reducing stunting and underweight rates over the past two decades, a high percentage of children remain affected, and there has been no progress on reducing wasting rates [3]. Around 25% of children under five years were stunted, 8% were wasted, and 19% were underweight according to the Nepal Demographic and Health Survey (NDHS) 2022. A study conducted in India [4] revealed that many factors, ranging from socio-economic to child feeding, to hygiene, are associated with the severe acute malnutrition (SAM) status of children. Similarly, in Nepal, factors including birth interval, child birth weight, sanitation and hygiene, birth order in the family, maternal age at birth, size of family, proper infant and young child feeding practices, gender inequality, socioeconomic status, and parental educational level were important determinants of malnutrition among children [5-9]. A previous study conducted by Adhikari RP et al. examined determinants of child malnutrition by analyzing NDHS 2006 to 2016 and highlighted risk factors for stunting, such as household wealth quintiles, mother’s years of schooling, child age, child size at birth, and child anemia [10]. Similarly, a study by Adhikari N et al. involving children up to 23 months of age focused on socio-demographic and household-level factors, such as wealth index, in addition to child-level factors including age, sex, child feeding practices, and breastfeeding, to explain factors affecting the nutritional status of infants and young children [11].

Considering all the known factors associated with nutritional status, appropriate weaning plays a major role in combating malnutrition in the most vulnerable children under two years. The World Health Organization has recommended the initiation of breastfeeding within one hour of birth, exclusive breastfeeding for the first six months, and introduction of nutritionally adequate and safe complementary food at six months together with continuing breastfeeding up to two years of age or beyond [12]. Inadequate timing, poor dietary diversity, and low meal frequency during weaning increase the risk of stunting, wasting, and underweight [13]. A previous study in Nepal [14] demonstrated that minimum dietary diversity and minimum acceptable diet were low among children aged 6-23 months, despite a high rate of timely introduction of solid, semi‐solid, or soft food and minimum meal frequency for both breastfed and non-breastfed children. Although a previous study reported that low maternal BMI, maternal education, wealth quintile, anemia, and low birth weight are significant predictors of childhood under-nutrition (stunting, wasting, and underweight) in 2022, child feeding practices/complementary feeding in multivariable models specific to each anthropometric outcome were not explored [15]. 

To fill this gap, the present study uses updated information from NDHS 2022 and aims to address child factors such as feeding practices, especially focusing on complementary feeding to younger children, along with other birth factors, parental factors, and socio-economic factors associated with all three nutritional indicators (stunting, wasting, and underweight). 

## Materials and methods

Study design

Secondary data analysis of Nepal Demographic and Health Survey (NDHS) 2022. NDHS is a nationally representative survey implemented by the Ministry of Health and Population, Nepal, under the Demographic and Health Surveys (DHS) program, which provides standardized and high-quality data on the health of the population, nutrition, and demographic indicators in low- and middle-income countries. The standardized questionnaires and rigorous sampling methods are used to collect DHS data, allowing for comparability across regions and over time. The NDHS dataset is available upon request, and permission to use the data was obtained from the DHS program [16].

Study population

**Figure 1:**
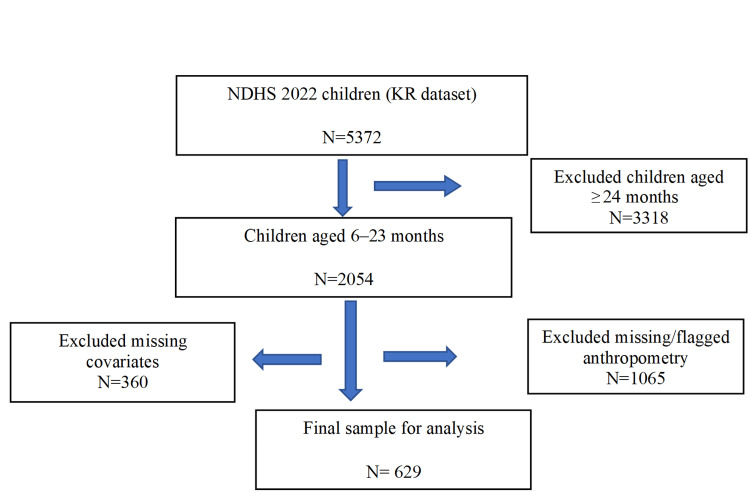
Flow Diagram for Study Sample (NDHS 2022) NDHS: Nepal Demographic and Health Survey; KR: Kids Recode

The initial NDHS 2022 dataset included all live births of the interviewed mothers within the five years preceding the survey. For the present analysis, the sample was restricted to children aged 6-23 months, consistent with WHO recommendations for the assessment of infant and young child feeding practices and child anthropometric outcomes. Children with valid anthropometric measurements (height/length and weight) required to compute height-for-age (HAZ), weight-for-height (WHZ), and weight-for-age (WAZ) z-scores based on WHO Child Growth Standards were included [17]. As shown in Figure 1, children with missing anthropometric values, as defined by DHS flag variables, were excluded. Additionally, children with missing data on key explanatory variables, including maternal, paternal, household, and feeding-related characteristics, were excluded from the analysis. After filtering children 6-23 months and excluding children whose anthropometric and other covariate data were missing, the total sample of 629 was retained in the final analysis.

Variables

Dependent variables

The three anthropometric indicators used to monitor the nutritional status of children were stunting (low Height-for-Age z-score, HAZ), wasting (low Weight-for-Height z-score, WHZ), and underweight (low Weight-for-Age z-score, WAZ). The WHO growth standards were used as the reference, and these indicators were coded as binary variables. From the NDHS data, variables HW70, HW71, and HW72 correspond to HAZ, WHZ, and WAZ, respectively.

Independent variables

Previous studies [18,19] showed an association between parental education and childhood under-nutrition in low- and middle-income countries. Regional variables were incorporated to capture geographic disparities in access to health services, food availability, and infrastructure [13, 15]. Feeding indicators, including minimum dietary diversity and feeding frequency, were included as they represent immediate determinants of nutritional status and have been shown to be significantly associated with child under-nutrition in multiple DHS-based studies [13,14]. 

For analyzing factors affecting nutritional status among children aged 6-23 months, this study considered several independent child-related variables, including age (HW1), which was recategorized as 6-11 months and 12-23 months; sex (B4); birth order (Bord), recategorized from an integer into ≤3 and ≥4; birth size (M18), categorized as very small, smaller than average, average, larger than average, or very large; and recent history of diarrhea (H11), recorded as yes or no.

Feeding frequency was recoded after adding up the total number of meals the child received by using variables from the NDHS data (children receiving meals ≥4 times were considered adequate, and ≤ 3 times were considered inadequate). The minimum dietary diversity score was calculated by adding up all the food items that were consumed by the child by using the food groups as provided as variables in the NDHS data (children consuming ≥5 food groups were considered to have met the criterion, and if ≤ 5 for those who did not). Feeding frequency and dietary diversity scores were calculated by referring to WHO guidelines for infant and young child feeding [12]. 

Maternal factors included age (V012: 15-24 years, 25-34 years, 35-49 years), education level (V106: no education, basic, secondary, higher), and BMI (V445: low BMI, normal, overweight, obese). Paternal education (V701) was recategorized after excluding the “don’t know” category and grouped as no education, basic, secondary, and higher. Household factors included wealth index (V190: poorest, poorer, middle, richer, richest) and regions (V101: all provinces).

Data analysis

Descriptive analysis was carried out to describe the background characteristics of children aged 6-23 months. To assess the factors related to child under-nutrition measures, adjusting for potential covariates, binomial logistic regression analysis was applied, which has also been applied in the previous studies using DHS data [9,10,20]. Jamovi software was used for statistical analysis [21].

## Results

Table 1 shows the descriptive statistics of the data used for the following analyses. Among the children, 145 (23.1%) were stunted, 65 (10.3%) were wasted, and 121 (19.2%) were underweight. There were more male children than female (n= 338; 53.7%). Most children belonged to the 12-23-month age group (n = 437; 69.5%). The majority of children were perceived to have an average birth size (n = 463; 73.6%). A higher number of children received meals four or more times a day (n=532; 84.6%). Feeding frequency was assessed only among breastfed children, as there were no non-breastfed children in the sample. More than half of the children did not meet the minimum dietary diversity score criteria (n = 335; 53.3%). Regarding maternal characteristics, 112 (17.8%) mothers had no formal education, while 225 (35.8%) and 269 (42.8%) had basic and secondary education, respectively. A few mothers were obese (n = 16; 2.5%). Among fathers, 248 (39.4%) had basic education, and 289 (45.9%) had secondary education. Most children belonged to households in the poorest (n = 210; 33.4%) and poorer (n = 142; 22.6%) wealth categories. 

**Table 1:** Descriptive presentation of children 6-23 months, mothers and household characteristics (N=629)

Variable	Category	n (%)
Nutritional status		
Stunting	No	484 (76.9)
	Yes	145 (23.1)
Wasting	No	564 (89.7)
	Yes	65 (10.3)
Underweight	No	508 (80.8)
	Yes	121 (19.2)
Child characteristics		
Sex (B4)	Male	338 (53.7)
	Female	291 (46.3)
Age (months)	6–11	192 (30.5)
	12–23	437 (69.5)
Recent diarrhea (last 2 weeks) (H11)	No	524 (83.3)
	Yes	105 (16.7)
Birth order (Bord)	Low birth order	562 (89.3)
	High birth order	67 (10.7)
Birth size (M18)	Very large	18 (2.9)
	Larger than average	68 (10.8)
	Average	463 (73.6)
	Smaller than average	60 (9.5)
	Very small	20 (3.2)
Feeding frequency (FF)	Adequate	532 (84.6)
	Inadequate	97 (15.4)
Minimum dietary diversity score (MDDS)	Not met criterion	335 (53.3)
	Met criterion	294 (46.7)
Maternal characteristics		
Mother’s education (V106)	No education	112 (17.8)
	Basic	225 (35.8)
	Secondary	269 (42.8)
	Higher	23 (3.7)
Maternal BMI (MBMI)	Low BMI	109 (17.3)
	Normal	409 (65.0)
	Overweight	95 (15.1)
	Obese	16 (2.5)
Mother’s age	15-24 years	317 (50.4)
	25-34 years	276 (43.9)
	35-49 years	36 (5.7)
Paternal characteristics		
Father’s education (V701)	No education	53 (8.4)
	Basic	248 (39.4)
	Secondary	289 (45.9)
	Higher	39 (6.2)
Wealth index (V190)	Poorest	210 (33.4)
	Poorer	142 (22.6)
	Middle	110 (17.5)
	Richer	99 (15.7)
	Richest	68 (10.8)
Region (V101)	Koshi	101 (16.1)
	Madhesh	113 (18.0)
	Bagmati	67 (10.7)
	Gandaki	42 (6.7)
	Lumbini	99 (15.7)
	Karnali	118 (18.8)
	Sudurpashchim	89 (14.1)

Factors associated with stunting

Table 2 shows the result of the multiple logistic regression analysis for stunting. Children with increasing age showed significantly higher odds of being stunted compared with those in the lower age group (OR = 3.77; 95% CI: 2.10-6.77; p < 0.001). Children with very small perceived birth size had more than six times higher odds of experiencing stunting compared with those with average birth size (OR = 6.06; 95% CI: 2.05-17.91; p = 0.001). Maternal education showed a strong protective association. Compared with mothers with no education, having basic, secondary and higher education had significantly lower odds of stunting ((OR = 0.46; 95% CI: 0.25-0.88; p = 0.018), (OR = 0.19; 95% CI: 0.09-0.39; p < 0.011) and (OR = 0.06; 95% CI: 0.01-0.58; p < 0.016)). Similarly, children born to mothers aged 25-34 years and 35-49 years had significantly lower odds of stunting compared with those born to younger mothers aged 15-24 years (OR = 0.58; 95% CI: 0.36-0.94; p = 0.026) and (OR = 0.26; 95% CI: 0.09-0.80; p = 0.018), respectively. Children from households in the poorer, middle, and richest wealth index had significantly lower odds of stunting compared with those from the poorest households (OR = 0.43; 95% CI: 0.23-0.80; p = 0.007) (OR = 0.42; 95% CI: 0.20-0.89; p = 0.023) and (OR = 0.22; 95% CI: 0.07-0.68; p = 0.008) respectively, while richer group was not statistically significant. Other variables, including child sex, recent diarrhea, feeding frequency, minimum dietary diversity score, and region, were not significant. 

**Table 2:** Binomial logistic regression analysis of factors associated with stunting Note. Estimates represent the log odds of "Stunting = 1" vs. "No Stunting = 0", odds ratios (OR) with 95% confidence intervals (CI). p < 0.05 was considered statistically significant. * provinces

Model Coefficients for Stunting (N= 629)						95% Confidence Interval	
Predictor	Estimate	SE	Z	p	OR	Lower	Upper
Intercept	-0.745	0.633	-1.177	0.239	0.475	0.137	1.641
Sex (B4), Ref: male							
Female	-0.156	0.22	-0.708	0.479	0.856	0.556	1.317
Child age, Ref: 6-11 months							
12-23 months	1.327	0.299	4.434	< 0.001	3.768	2.096	6.774
Recently had diarrhea (H11), Ref: No diarrhea							
Yes, within the last two weeks	-0.167	0.299	-0.558	0.577	0.846	0.471	1.52
Birth order (Bord), Ref: low birth order							
High birth order	0.625	0.372	1.681	0.093	1.867	0.901	3.868
Birth size (M18), Ref: average							
Very large	-1.379	0.854	-1.616	0.106	0.252	0.047	1.342
Larger than average	-0.548	0.375	-1.463	0.143	0.578	0.277	1.204
Smaller than average	0.217	0.351	0.617	0.537	1.242	0.624	2.474
Very small	1.802	0.553	3.258	0.001	6.059	2.05	17.91
Feeding frequency (FF), Ref: adequate							
Inadequate	0.092	0.327	0.281	0.778	1.096	0.578	2.079
Minimum dietary diversity score (MDDS), Ref: not met criterion							
Met criterion	0.317	0.238	1.329	0.184	1.373	0.86	2.191
Maternal BMI (MBMI), Ref: normal							
Low BMI	0.385	0.277	1.391	0.164	1.469	0.854	2.527
Overweight	0.08	0.355	0.226	0.821	1.084	0.541	2.172
Obese	-0.657	1.108	-0.593	0.553	0.519	0.059	4.548
Mother’s education (V106), Ref: no education							
Basic	-0.768	0.325	-2.366	0.018	0.464	0.245	0.877
Secondary	-1.682	0.379	-4.443	< 0.001	0.186	0.089	0.391
Higher	-2.907	1.202	-2.419	0.016	0.055	0.005	0.576
Mother's age, Ref: 15-24 years							
25-34 years	-0.55	0.248	-2.22	0.026	0.577	0.355	0.938
35-49 years	-1.342	0.567	-2.365	0.018	0.261	0.086	0.795
Father's education (V701), Ref: no education							
Basic	0.076	0.4	0.19	0.849	1.079	0.493	2.361
Secondary	0.304	0.429	0.707	0.48	1.355	0.584	3.143
Higher	0.849	0.75	1.132	0.258	2.338	0.537	10.175
Wealth index (V190), Ref: poorest							
Poorer	-0.844	0.315	-2.675	0.007	0.43	0.232	0.798
Middle	-0.865	0.381	-2.268	0.023	0.421	0.199	0.889
Richer	-0.661	0.376	-1.758	0.079	0.516	0.247	1.079
Richest	-1.53	0.58	-2.637	0.008	0.217	0.069	0.675
Regions* (V101), Ref: Bagmati							
Koshi	-0.958	0.507	-1.891	0.059	0.384	0.142	1.035
Madhesh	0.073	0.476	0.153	0.878	1.076	0.423	2.737
Gandaki	0.378	0.534	0.708	0.479	1.459	0.513	4.152
Lumbini	0.286	0.459	0.624	0.533	1.332	0.542	3.273
Karnali	0.091	0.455	0.199	0.842	1.095	0.449	2.67
Sudurpashchim	-0.849	0.512	-1.658	0.097	0.428	0.157	1.167

Factors associated with wasting

Table 3 shows the result of the multiple logistic regression analysis for wasting. Children who were reported as having larger than average birth size were significantly less likely to be wasted compared with those of average birth size (OR = 0.19; 95% CI: 0.04-0.87; p = 0.033). However, there were no statistically significant associations for children perceived as very large, smaller than average or very small at birth. Maternal nutritional status was strongly associated with wasting. Children whose mothers had normal BMI had significantly lower odds of wasting compared with children of low BMI mothers (OR = 3.61; 95% CI: 1.88-6.92; p < 0.001). Children whose mothers had secondary education showed significantly lower odds of wasting compared with the children of mothers with no education (OR = 0.27; 95% CI: 0.10-0.72; p = 0.009). There were no significant associations detected for basic and higher maternal education. Interestingly, children whose fathers had a secondary and higher education had significantly greater odds of wasting compared with those whose fathers had no education (OR = 3.56; 95% CI: 1.08-11.82; p = 0.038), (OR = 11.40; 95% CI: 2.14-60.58; p = 0.004). No significant associations were observed for child age, sex, recent diarrhea, feeding frequency, minimum dietary diversity score, wealth, and regions. 

**Table 3:** Binomial logistic regression analysis of factors associated with wasting Note. Estimates represent the log odds of "Wasting = 1" vs. "No Wasting = 0", odds ratios (OR) with 95% confidence intervals (CI). p < 0.05 was considered statistically significant. * provinces

Model Coefficients; Wasting (N=629)						95% Confidence Interval	
Predictor	Estimate	SE	Z	p	OR	Lower	Upper
Intercept	-2.761	0.859	-3.212	0.001	0.063	0.012	0.341
Sex (B4), Ref: male							
Female	-0.396	0.297	-1.333	0.183	0.673	0.376	1.205
Child age, Ref: 6-11 months							
12-23 months	0.2	0.347	0.577	0.564	1.222	0.619	2.412
Recently had diarrhea (H11), Ref: No diarrhea							
Yes, last two weeks	0.514	0.347	1.482	0.138	1.672	0.847	3.299
Birth order (Bord), Ref: low birth order							
High birth order	0.852	0.478	1.783	0.075	2.345	0.919	5.982
Birth size (M18), Ref: average							
Very large	0.173	0.876	0.198	0.843	1.189	0.214	6.616
Larger than average	-1.681	0.788	-2.132	0.033	0.186	0.04	0.873
Smaller than average	0.518	0.428	1.208	0.227	1.678	0.725	3.884
Very small	0.372	0.641	0.58	0.562	1.451	0.413	5.099
Feeding frequency (FF), Ref: adequate							
Inadequate	0.576	0.377	1.527	0.127	1.779	0.849	3.725
Minimum dietary diversity score (MDDS), Ref: not met criterion							
Met criterion	-0.028	0.325	-0.087	0.931	0.972	0.514	1.839
Maternal BMI (MBMI), Ref: normal							
Low BMI	1.284	0.332	3.87	<0.001	3.611	1.884	6.92
Overweight	-0.054	0.494	-0.11	0.913	0.947	0.36	2.493
Obese	-14.487	927.77	-0.016	0.988	0	0	Inf
Mother’s education (V106), Ref: no education							
Basic	-0.44	0.434	-1.015	0.31	0.644	0.275	1.506
Secondary	-1.299	0.497	-2.614	0.009	0.273	0.103	0.722
Higher	-1.441	1.008	-1.43	0.153	0.237	0.033	1.707
Mother's age, Ref: 15-24 years							
25-34 years	0.113	0.322	0.351	0.726	1.12	0.595	2.106
35-49 years	-0.818	0.724	-1.13	0.258	0.441	0.107	1.824
Father's education (V701), Ref: no education							
Basic	0.157	0.577	0.272	0.785	1.17	0.378	3.623
Secondary	1.271	0.612	2.077	0.038	3.564	1.075	11.82
Higher	2.433	0.852	2.854	0.004	11.395	2.143	60.582
Wealth index (V190), Ref: poorest							
Poorer	0.113	0.417	0.27	0.787	1.119	0.494	2.534
Middle	-0.078	0.491	-0.158	0.874	0.925	0.353	2.424
Richer	-0.328	0.518	-0.633	0.527	0.72	0.261	1.988
Richest	-0.286	0.629	-0.455	0.649	0.751	0.219	2.577
Regions* (V101), Ref: Bagmati							
Koshi	-0.728	0.708	-1.028	0.304	0.483	0.121	1.935
Madhesh	-0.143	0.652	-0.22	0.826	0.867	0.242	3.108
Gandaki	0.059	0.75	0.079	0.937	1.061	0.244	4.615
Lumbini	0.885	0.6	1.474	0.141	2.423	0.747	7.858
Karnali	-0.591	0.679	-0.87	0.384	0.554	0.146	2.096
Sudurpashchim	-0.216	0.641	-0.336	0.737	0.806	0.229	2.834

Factors associated with under-nutrition

Table 4 shows the result of the multiple logistic regression analysis for underweight. Children whose birth size was reported as very small were showing significantly higher odds of being underweight compared with those of average birth size (OR = 6.28; 95% CI: 2.06-19.17; p = 0.001). Similarly, children of mothers with normal BMI had more than two times less odds of being underweight compared with children of lower BMI mothers (OR = 2.31; 95% CI: 1.36-3.93; p = 0.002). Maternal education showed a negative association with underweight. Children whose mothers had secondary level education showed significantly lower odds of being underweight as compared to children from uneducated mothers (OR = 0.29; 95% CI: 0.14-0.62; p = 0.001). Several variables showed borderline significance with underweight. Children in the 12-23 months group have higher odds of being underweight than younger children 6-11 months (p = 0.092). Recent diarrheal illness was also marginally associated with increased odds of underweight (p = 0.094). Similarly, children born to younger mothers, 15-24 years, had higher odds of being underweight compared with those born to mothers aged 35-49 years (p = 0.070). Other covariates, including child sex, maternal age, paternal education, and household wealth, were not significantly associated with underweight, although Lumbini province showed a borderline association (p = 0.064). 

**Table 4:** Binomial logistic regression analysis of factors associated with underweight children Note. Estimates represent the log odds of "Underweight = 1" vs. "No Underweight = 0", odds ratios (OR) with 95% confidence intervals (CI). p < 0.05 was considered statistically significant.

Model Coefficients; Underweight (N=629)						95% Confidence Interval	
Predictor	Estimate	SE	Z	p	OR	Lower	Upper
Intercept	-1.732	0.686	-2.523	0.012	0.177	0.046	0.679
Sex (B4), Ref: male							
Female	0.186	0.228	0.816	0.414	1.204	0.771	1.881
Child age, Ref: 6-11 months							
12-23 months	0.461	0.274	1.685	0.092	1.586	0.928	2.713
Recently had diarrhea (H11), Ref: No diarrhea							
Yes, last two weeks	0.476	0.285	1.673	0.094	1.610	0.921	2.814
Birth order (Bord), Ref: low birth order							
High birth order	0.504	0.388	1.298	0.194	1.656	0.773	3.545
Birth size (M18), Ref: average							
Very large	-0.553	0.850	-0.651	0.515	0.575	0.109	3.044
Larger than average	-0.423	0.404	-1.048	0.295	0.655	0.297	1.445
Smaller than average	0.499	0.351	1.421	0.155	1.647	0.828	3.277
Very small	1.837	0.569	3.227	0.001	6.279	2.057	19.168
Feeding frequency (FF), Ref: adequate							
Inadequate	0.115	0.319	0.362	0.718	1.122	0.601	2.096
Minimum dietary diversity score (MDDS), Ref: not met criterion							
Met criterion	-0.189	0.253	-0.749	0.454	0.828	0.504	1.358
Maternal BMI (MBMI), Ref: normal							
Low BMI	0.836	0.271	3.085	0.002	2.307	1.356	3.925
Overweight	-0.359	0.404	-0.888	0.374	0.698	0.316	1.543
Obese	-14.787	530.946	-0.028	0.978	0.000	0.000	Inf
Mother’s education (V106), Ref: no education							
Basic	-0.488	0.337	-1.450	0.147	0.614	0.317	1.187
Secondary	-1.237	0.389	-3.177	0.001	0.290	0.135	0.623
Higher	-2.058	1.153	-1.786	0.074	0.128	0.013	1.222
Mother's age, Ref: 15-24 years							
25-34 years	0.152	0.252	0.603	0.547	1.164	0.711	1.905
35-49 years	-1.171	0.648	-1.809	0.070	0.310	0.087	1.103
Father's education (V701), Ref: no education							
Basic	-0.331	0.411	-0.805	0.421	0.718	0.321	1.608
Secondary	0.472	0.440	1.075	0.283	1.604	0.678	3.796
Higher	0.533	0.728	0.732	0.464	1.703	0.409	7.091
Wealth index (V190), Ref: poorest							
Poorer	-0.191	0.325	-0.587	0.557	0.826	0.437	1.563
Middle	-0.251	0.383	-0.654	0.513	0.778	0.367	1.649
Richer	-0.255	0.397	-0.643	0.520	0.775	0.356	1.688
Richest	-0.831	0.554	-1.500	0.134	0.436	0.147	1.290
Regions* (V101), Ref: Bagmati							
Koshi	-0.336	0.583	-0.575	0.565	0.715	0.228	2.243
Madhesh	0.754	0.544	1.387	0.165	2.126	0.732	6.174
Gandaki	0.841	0.617	1.364	0.173	2.319	0.693	7.769
Lumbini	0.978	0.529	1.849	0.064	2.659	0.943	7.498
Karnali	-0.015	0.557	-0.027	0.979	0.985	0.330	2.938
Sudurpashchim	-0.144	0.574	-0.251	0.802	0.866	0.281	2.664

## Discussion

This study assessed factors associated with nutritional status among 6-23-month-old children who were selected from the NDHS 2022 data. The result of this study shows that perceived child age, birth size, maternal education, maternal age, and household wealth are statistically significant factors for stunting. Perceived birth size, maternal BMI, maternal education, and paternal education are significant factors for wasting. In this study, birth size and maternal factors such as BMI, education, and age are significant factors for under-nutrition.

This study showed that the increasing age of children was significantly associated with stunting. Previous studies, such as [9,22], also supported our results. This might be because stunting is reflected as chronic malnutrition, which can be seen after long-term nutritional deficiencies, whereas wasting is a result of acute under-nutrition. The children who were perceived to be smaller at birth showed a higher probability of stunting, wasting, and underweight as compared to children who were perceived to be bigger. This finding is consistent with other studies, such as [9,20]. The result of this study demonstrates that mothers with a higher education level have a lower probability of their children getting stunting, wasting, and being underweight. It is consistent with the findings from the previous studies conducted in Ethiopia [20], Bangladesh [23], Uganda [24], and Nepal [9]. The reasons might be that educated mothers are more aware of the child care and feeding practices, proper hygiene, greater ability to utilize health care facilities, etc. [25-28].

Unexpectedly, children from fathers with higher education showed higher chances of being wasted than children from fathers with no education. Similar to our finding, a study that performed multi-country analysis in low- and middle-income settings found that higher paternal education was associated with increased odds of certain forms of the double burden of malnutrition compared with no paternal education [18]. Higher education in fathers may be associated with employment patterns that reduce time spent on child care or supervision, particularly in contexts where maternal roles in feeding and caregiving are dominant. Previous studies supporting our finding indicated that maternal education is actually more important for reducing childhood under-nutrition than paternal education [18]. Additionally, the present study also shows that children of fathers with higher education were less likely to be stunted but more likely to be wasted, suggesting these children tend to be taller yet relatively thinner. A divergence between chronic and acute forms of under-nutrition can be assumed. This study demonstrates that children from younger mothers (15-24 years) were more likely to be stunted and underweight than children from older mothers.

The present study also showed that maternal body mass index (BMI) is one of the major determinants of wasting and being underweight, whereas it showed no association with stunting. The children of mothers with normal to higher BMI showed less possibility of getting wasting and underweight than the children of mothers with low BMI. Other studies showed that higher maternal BMI had less risk of stunting and wasting [15,29]. Similarly, household wealth demonstrated a strong association with stunting but not with wasting and being underweight. Consistent with the result from this study, several studies [9,30] have reported findings indicating that wealth enhances food security, dietary diversity, healthcare utilization, sanitation, and overall living conditions, thereby reducing chronic under-nutrition risk.

Feeding frequency and minimum dietary diversity score were expected to be significant predictors of under-nutrition among children aged 6-23 months; however, no significant association was observed. Although these did not show a significant association in this analysis, the weaning period is a critical period for child growth and development. The findings from this study suggest that maternal and household factors strongly influence complementary feeding practices, highlighting the need for integrated nutrition interventions that support both caregivers and children during the weaning period.

Results from this study can be generalized to address populations with similar characteristics and would be useful in reviewing and designing new intervention strategies for further public health research focusing on child malnutrition.

The cross-sectional nature of the NDHS data limits causal inference between identified factors and nutritional outcomes. Perceived birth size may potentially hold recall bias. Furthermore, information on dietary intake and feeding practices was limited to short recall periods and may not fully capture habitual feeding patterns.

## Conclusions

The findings of this study showed that the increasing age of children was significantly associated with stunting. Maternal education, maternal age, and maternal BMI were important protective determinants of child under-nutrition. From the findings of this study, complementary feeding practices along with the maternal and household factors may help to minimize child under-nutrition. Strengthening maternal education, improving household economic conditions, delaying early motherhood, improving birth outcomes, and integrating nutrition-sensitive interventions during the weaning period are essential to improve child nutritional outcomes in Nepal.
